# State of health estimation for lithium-ion batteries based on differential thermal voltammetry features and particle swarm optimization-gated recurrent unit

**DOI:** 10.1371/journal.pone.0342942

**Published:** 2026-04-21

**Authors:** Bing Han, Xiaohan Sun, Youxin Wang, Yuting Song, Enguang Hou, Jiangang Wang, Yanliang Xu

**Affiliations:** 1 School of Rail Transportation, Shandong Jiao Tong University, Jinan, China; 2 School of Electrical Engineering, Shandong University, Jinan, China; 3 Jinan Park Development Service Center, Jinan, China; Sunway University, MALAYSIA

## Abstract

To address the lack of physical interpretability and weak model generalization in purely data-driven methods for estimating the State of Health (SOH) of LIBs, this study proposes a lithium battery SOH estimation method based on Differential Temperature- Voltammetry (DTV) characteristics and Particle Swarm Optimization-Gated Recurrent Unit (PSO-GRU). To capture thermodynamic characteristics during battery aging, First, this paper computes DTV curves based on thermo-electrical coupling responses during charge-discharge cycles and extracts a 6-dimensional physical feature vector to quantify Multiphysics evolution patterns in battery aging. Second, a PSO-GRU prediction model is established, employing the particle swarm optimization (PSO) algorithm to adaptively optimize the hyperparameters of the gated recurrent unit (GRU). Additionally, to address the issue of significant data fluctuations in the early stages of battery aging that interfere with long-term trend prediction, a training set optimization method based on aging stage segmentation is proposed. Simulation experiments demonstrate that this method achieves significantly higher SOH estimation accuracy on the NASA battery dataset compared to standard GRU and long short-term memory (LSTM) models. After applying the optimization strategy, the model’s mean absolute error (MAE) on the test set decreased from 1.45% to 0.75%, and the root mean square error (RMSE) decreased from 1.86% to 0.97%, demonstrating enhanced generalization capability and robustness. The experimental results validate the necessity of excluding non-stationary data from the formation period for constructing high-accuracy, long-term prediction models, providing new insights for the engineering application of data-driven methods.

## Introduction

Lithium-ion batteries (LIBs), as core components of new energy vehicles and energy storage systems, require precise State of Health (SOH) estimation as a critical function of battery management systems (BMS). Achieving robust estimation throughout the entire battery lifecycle remains a significant challenge [1,2] due to the complex internal physicochemical evolution involved in battery aging and its dependence on multi-physics coupling effects such as temperature and rate conditions [3–5]. Currently, SOH estimation primarily relies on two approaches: model-driven and data-driven. Among model-driven methods, equivalent circuit models (ECMs) are the most widely applied [6–8]. However, existing reviews and studies [9–11] indicate that such models heavily depend on parameter identification under specific operating conditions. They struggle to accurately track complex nonlinear parameter drift throughout the aging cycle, leading to reduced robustness after prolonged use. In contrast, data-driven approaches, exemplified by deep learning algorithms such as Recurrent Neural Networks (RNN) and Long Short-Term Memory (LSTM) networks, demonstrate robust nonlinear fitting capabilities [12–14]. To further improve estimation performance, recent studies have introduced advanced architectures, including Parallel Feature Fusion Networks (PFFN) [15], Internal Cascaded Neuromorphic Computing Systems (ICNCS) [16], and Time-Frequency Hybrid Neuromorphic Computing architectures [17], aiming to mine deep information from battery data. Yet, related literature [18–20] points out that purely data-driven models often lack sufficient physical interpretability, relying solely on statistical mapping relationships between data points. When test data distributions diverge from training data distributions, the model’s generalization capability and reliability become questionable.

Aiming to bridge this gap, hybrid modeling approaches integrating physical features have emerged as a research focus. These methods aim to synergistically leverage the interpretability of physical mechanisms with the nonlinear mapping capabilities of data-driven models. Some studies [21–23] employed Incremental Capacity Analysis (ICA) to extract features, constructing hybrid SOH estimation models that enhanced interpretability. Other researchers [24–26] further investigated Differential Voltage Analysis (DVA), quantitatively tracking aging patterns like Loss of Lithium Inventory (LLI) by decoupling DVA feature peaks.

Although these studies advanced electrical-level understanding, they neglected thermal characteristics. Dependence exclusively on voltage analysis proves insufficient to fully decouple the complex electrochemical-thermodynamic evolution during aging. Recent research [27–30] introduced differential temperature voltammetry (DTV) analysis. By leveraging the thermo-electrical coupling during electrode material intercalation and deintercalation, this method successfully captured characteristic signals strongly correlated with aging states. These studies indicate that peak position drift in DTV curves directly characterizes the increased internal resistance and enhanced polarization effects caused by battery aging, while peak height evolution effectively reveals degradation mechanisms related to internal entropy changes. Compared to the traditional ICA method employed by related research [21], DTV provides supplementary phase-transition information with a high signal-to-noise ratio, independent of differential voltage measurements, thereby offering a new dimension for enhancing model robustness under complex operating conditions.

Based on the analysis above, this paper constructs an SOH estimation framework integrating DTV physical mechanisms with a globally optimized deep learning network. This approach directly extracts thermo-electric coupling features from DTV curves, effectively quantifying the thermodynamic and impedance evolution states within the battery while avoiding the noise associated with complex differential operations. To mitigate the influence of data quality on model performance, a data dynamic screening strategy based on aging stages is designed to eliminate atypical fluctuations caused by battery activation or unstable electrochemical behavior during the early aging phase, thereby optimizing the training dataset. Building upon this foundation, a particle swarm optimization (PSO) algorithm is introduced to perform global hyperparameter optimization for the gated recurrent unit (GRU), overcoming the limitations of manual parameter tuning in traditional networks. This ultimately achieves high-precision, highly robust tracking of SOH degradation trajectories.

## 1. Feature extraction

This chapter employs the DTV method to analyze the battery aging process and extracts six health features strongly correlated with SOH.

### 1.1 Battery aging data

All research in this paper is based on the publicly available LIB dataset from the NASA Ames Prognostics Center of Excellence (PCoE) [31]. The experimental subject is a commercial 18650 LIB with a nominal capacity of 2Ah, featuring a nickel-cobalt-manganese (NCM) cathode material and a graphite anode material. The SOH of a battery is typically defined as the ratio of the current maximum available capacity to the nominal capacity, calculated as follows:


$$SOH=\frac{C_{curr}}{C_{nom}}\times 100\%$$ (1)

Where *C*_*curr*_ represents the maximum available capacity during the current cycle, and *C*_*nom*_ denotes the battery’s factory-specified nominal capacity (2.0 Ah in this study). Given that *C*_*nom*_ is constant, SOH exhibits a linear relationship with *C*_*curr*_. Therefore, this study directly employs battery capacity as the SOH metric and evaluates model performance using capacity prediction error.

All cycling and aging tests for this dataset were conducted at a constant room temperature of 24°C. The charging process employed a constant current-constant voltage (CC-CV) mode: first charging at a constant current of 1.5A until reaching 4.2V, then switching to constant voltage charging until the current dropped to 20mA. The discharge process employs a 2A constant current mode until the voltage drops to 2.7V.

**Fig 1:**
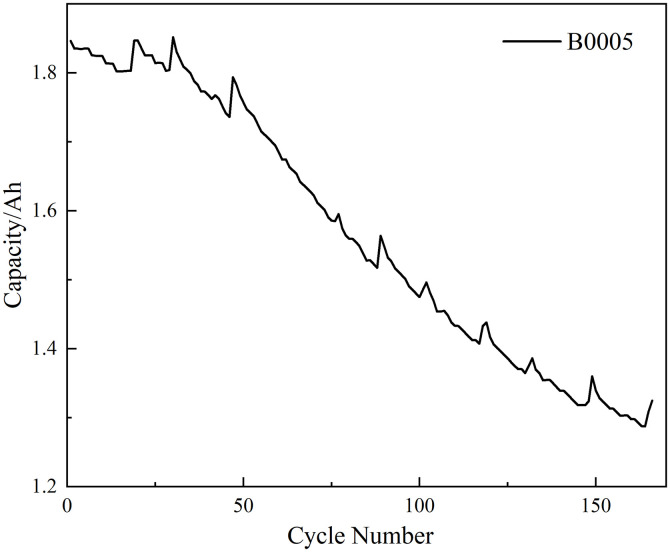
Capacity fade curve of battery B0005.

Battery B0005 was selected as the subject of this study. This battery underwent a complete aging process from new to end-of-life (capacity degraded to 1.4Ah). After removing abnormal cycles unsuitable for feature extraction, 166 valid cycles with complete constant-current charging phases were ultimately retained as the raw data foundation for constructing subsequent health feature vectors. As shown in Fig 1, the capacity fade curve of the B0005 battery exhibits complex nonlinear characteristics accompanied by localized capacity recovery phenomenon. This provides a high-quality yet challenging data foundation for subsequent health feature extraction and long-term SOH prediction research.

### 1.2 Calculation of DTV curves and health feature extraction

This paper proposes a health feature extraction framework based on DTV curves. The physical basis of this method is grounded in the entropic heat effect of LIBs. During charging and discharging, lithium ions undergo intercalation and deintercalation within the electrode materials (graphite anodes and NCM cathodes in this study), causing periodic changes in the lattice ordering—specifically, entropy changes. According to thermodynamic principles, these entropic changes manifest as the absorption or release of energy in the form of reversible heat, inducing minute temperature fluctuations in the battery that are closely correlated with electrochemical phase transitions [32,33].

By calculating the derivative of temperature with respect to voltage (dT/dV), the DTV analysis amplifies and characterizes these subtle thermal signals driven by electrode phase transitions. Consequently, the peak and valley profile of the DTV curve can be regarded as a “thermal fingerprint” of internal electrochemical processes. The evolution of their positions and amplitudes directly reflects key aging mechanisms, such as Loss of Active Material (LAM) and LLI. This provides a theoretical basis for extracting physically meaningful health features from the perspective of thermo-electric coupling.

Notably, compared to traditional ICA or DVA methods, the DTV approach demonstrates dual advantages. Regarding signal robustness, ICA (*dQ/dV*) and DVA (*dV/dQ*) heavily rely on voltage or capacitance differentials, making them highly susceptible to sampling noise interference. DTV, however, leverages the battery’s thermal inertia. Temperature response inherently lags behind voltage fluctuations, endowing DTV with inherent low-pass filtering properties that effectively suppress high-frequency measurement noise. Regarding physical mechanisms, ICA solely reflects the electrode’s charge storage capacity, whereas DTV correlates *dT/dV* with entropy-heat effects, directly revealing the thermodynamic essence of electrochemical reactions. Lattice structure distortion and phase transitions caused by battery aging are often accompanied by significant entropy changes. Consequently, DTV captures these minute thermal signatures directly linked to alterations in lattice order, providing richer physical insights for identifying aging mechanisms than purely electrical methods.

The practical value of this framework lies in its potential to construct “gray-box” SOH estimation models with significantly improved prediction accuracy and robustness. This is achieved by providing machine learning models with information-rich and physically interpretable inputs. The DTV feature extraction process primarily involves DTV curve calculation, smoothing, and the identification and extraction of key features.

#### 1.2.1 Calculation and smoothing of DTV curves.

The DTV curve based on the surface temperature of the LIB is obtained through the following steps:

Step 1: Measure the surface temperature T and terminal voltage V during the constant-current (CC) charging phase of the LIB.
Step 2: Calculate the DTV curve. The DTV is defined as the derivative of battery temperature with respect to terminal voltage, expressed as Eq (2):

$$DTV=\frac{dT/dt}{dV/dt}=\frac{dT}{dV}$$ (2)

Where *T* denotes the battery surface temperature, *V* denotes the terminal voltage, and *t r*epresents the sampling time. To minimize noise interference in the calculation, a finite difference method using two sampling points separated by a specific interval is employed to approximate the DTV value at time *k*. A larger sampling interval effectively suppresses noise but risks losing critical features; conversely, an interval that is too small fails to filter out measurement noise, resulting in instability. Based on extensive experimental comparisons to balance noise suppression and feature preservation, a differential interval of *N/*15 (where *N* is the total number of sampling points) was selected. Given that practical sampling data is discrete and noisy, direct differentiation would result in severe fluctuations. Therefore, a finite difference approximation is applied, as defined in Eq (3):


$$DTV=\frac{T\left(k\right)-T\left(k-N/15\right)}{V\left(k\right)-V\left(k-N/15\right)}$$ (3)

where *T*(*k*) and *V*(*k*) represent the temperature and voltage at time step *k*, respectively, and *N* denotes the total number of sampling points in the charging phase.

**Fig 2:**
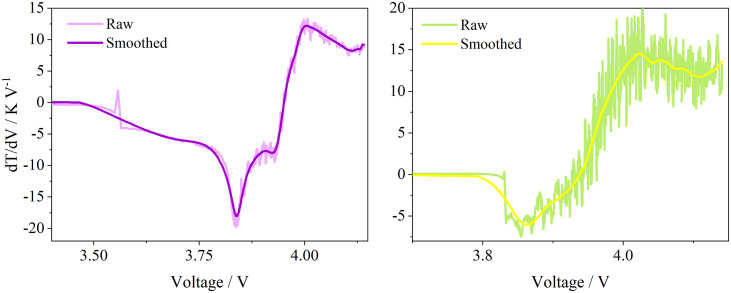
DTV curves of the 7th and 67th cycles.


Step 3: Smooth the DTV curve. Raw measurement data typically contains substantial noise, and obtaining a smooth DTV curve is critical for accurate SOH estimation. As shown in Fig 2, the raw DTV curves for the 7th and 67th cycles exhibit severe fluctuations due to noise, which significantly hinders the extraction of aging features. Therefore, the Savitzky-Golay (S-G) filter is employed for denoising. As a polynomial least-squares smoothing filter, the S-G filter is highly suitable for this application. Its primary advantage lies in preserving the shape and height of waveform peaks while filtering noise, ensuring that key aging-related features (e.g., peak/valley positions and amplitudes) remain undistorted. While the Mean Squared Error (MSE) is a common metric for evaluating smoothing effects, solely minimizing MSE might lead to “flattening” or shifting of peaks if the window size is too large. To balance denoising performance and feature fidelity, various parameter combinations were evaluated. The combination that yielded the smoothest trajectories for features *F*_1_ – *F*_6_ while maintaining clear physical trends was selected. Consequently, the optimal parameters were set as follows: a polynomial order of 3 and a sliding window length of 121. The processed curve became smooth while clearly preserving its core “two-valley, one-peak” profile. This smoothing process was applied to all cycle data, laying the foundation for constructing robust health feature vectors. The fundamental equation of the S-G filter is given by Eq (4):

$$y\left(i\right)=\sum_{j=-m}^{j=m}\frac{\text{1}}{N}C_{j}x(i+j)$$ (4)

Where *x*(*i*) represents the original signal; *y*(*i*) denotes the smoothed output signal; *C*_*j*_ is the convolution coefficient of the S-G filter; and the window width is 2*m* + 1, where *m* is the half-width of the smoothing window.

#### 1.2.2 Construction of eigenvectors.

**Fig 3:**
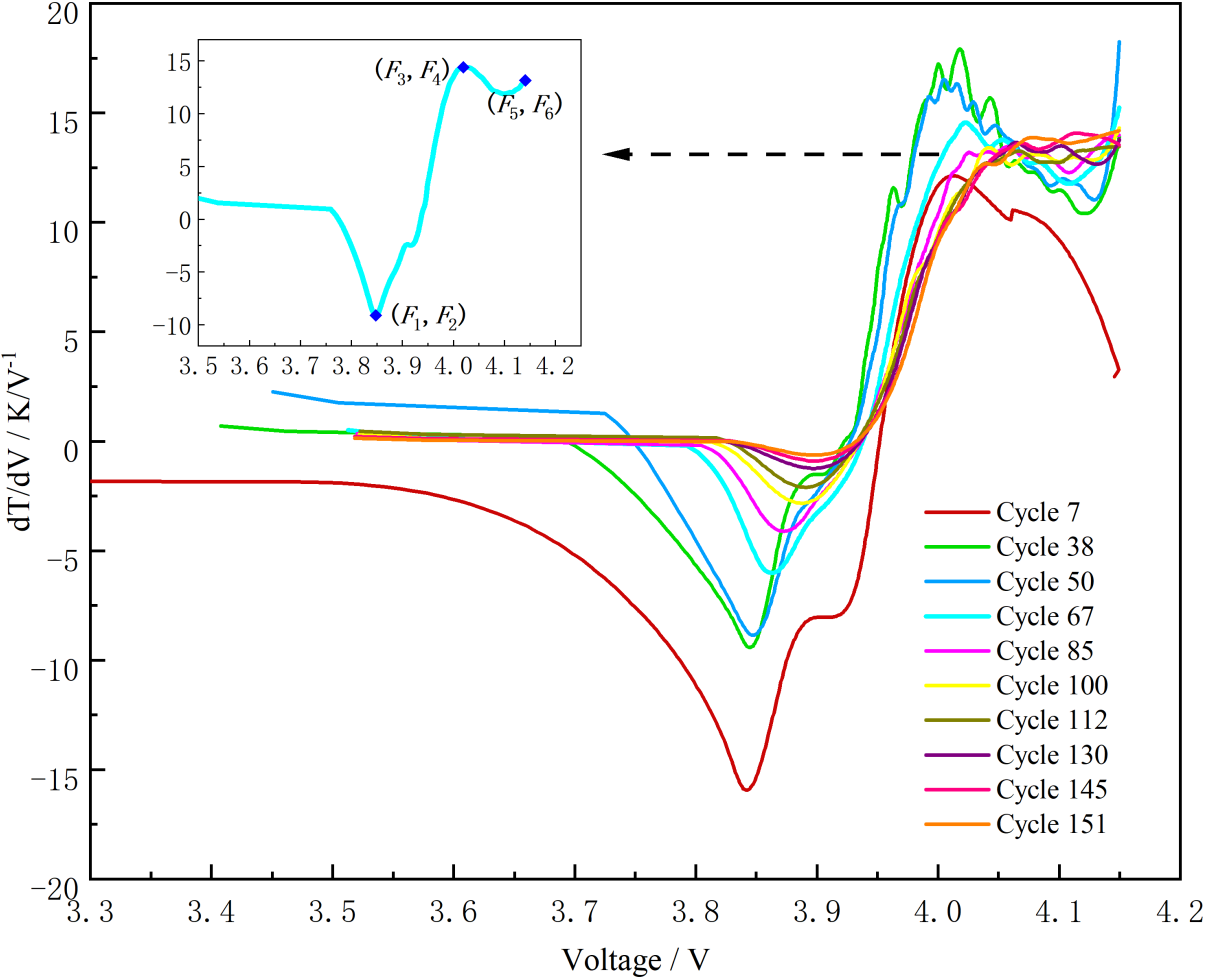
DTV curves of battery B0005 at different aging stages.

Fig 3 displays ten representative DTV curves selected from different aging stages. It can be observed that the DTV curve exhibits a distinct “two-valley, one-peak” morphology, and the evolution of these peaks and valleys follows a discernible trend during battery aging.

This distinctive morphology is closely related to the electrochemical phase transition processes occurring in the electrode materials during charging. Specifically, for the NCM/graphite system used in this study—where graphite serves as the anode—these characteristic peaks and valleys are primarily interpreted as reflecting the staged phase transitions of the graphite anode during lithium intercalation [34,35]. As the battery ages, the LLI and LAM alter the kinetic and thermodynamic properties of these phase transitions. Macroscopically, this manifests as a drift in the positions of the DTV peaks and valleys (*F*_1_*, F*_3_*, F*_5_) and an attenuation in their amplitudes (*F*_2_*, F*_4_*, F*_6_). Therefore, the six-dimensional vector formed by these features can be regarded as a comprehensive “thermal fingerprint” of the battery’s internal health state.

As shown in Fig 3, the peak amplitude gradually decreases with aging, while its voltage position exhibits an increasing trend. Conversely, the valley amplitudes gradually increase (become less negative). The voltage position of the first valley shows an increasing trend, whereas that of the second valley exhibits a decreasing trend. Based on these evolutionary trends, the positions of the two valleys and one peak are identified using local extrema detection. Their corresponding voltage values and DTV amplitudes are recorded to construct a six-dimensional health feature vector. The extracted Valley1, Peak1, and Valley2 correspond to (*F*_1_*, F*_2_), (*F*_3_*, F*_4_), and (*F*_5_*, F*_6_), respectively, as defined in the inset of Fig 3. The mathematical expressions for peak and valley extraction are given by Eq (5) and Eq (6):


$$\begin{array}{c} V_{\text{peak}}=V_{i}{|}_{\frac{dD}{dV}=0,\text{\textbackslash{}hspace\{0.17em}}f\left(V_{i}\right)\geq f\left(V_{k}\right),\text{\textbackslash{}hspace\{0.17em}\}V_{k}\in \left(V_{i-1},V_{i+1}\right)\}\\ D_{\text{peak}}\;=g\left(V_{\text{peak}}\right) \end{array}$$ (5)

$$\begin{array}{c} V_{\text{valley}}=V_{i}{|}_{\frac{dD}{dV}=0,\text{\textbackslash{}hspace\{0.17em}}f\left(V_{i}\right)\leq f\left(V_{k}\right),\text{\textbackslash{}hspace\{0.17em}\}V_{k}\in \left(V_{i-1},V_{i+1}\right)\}\\ D_{\text{valley}}\;=g\left(V_{\text{valley}}\right) \end{array}$$ (6)

where the DTV value corresponds to the battery voltage, and *V*_*i-*1_ and *V*_*i+*1_ represent the sampled voltages at the preceding and subsequent sampling points, respectively. When applying the aforementioned method for full-lifecycle feature extraction, this study identified a very small number of cycles (three in total) where measurement noise caused severe distortion of the DTV curve morphology. These cycles lacked the essential “two valleys and one peak” structure required for constructing feature vectors, rendering them incapable of yielding valid solutions via Eq (5) and Eq (6). To ensure the validity and consistency of the model input data, this study excluded these anomalous cycles that could not form complete feature vectors. Ultimately, 166 valid cycles were retained as the experimental dataset.

**Fig 4:**
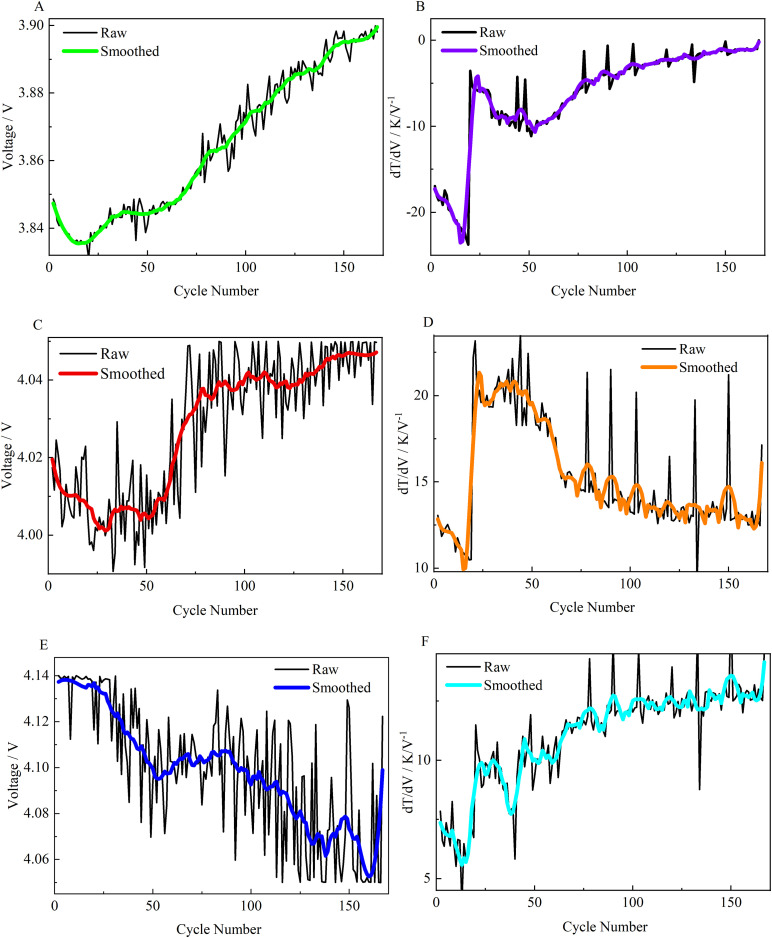
Evolutionary trajectories of six-dimensional features *F*_1_*-F*_6_. (A, C, E) The evolution trends of voltage-related features (*F*_1_*, F*_3_*, F*_5_). (B, D, F) The evolution trends of DTV-related features (*F*_2_*, F*_4_*, F*_6_). The colored solid lines represent the data processed by the S-G smoothing filter, while the black lines represent the raw data.

After constructing the health feature vector spanning the entire lifecycle, we observed that due to random factors such as measurement noise, the evolutionary trajectories of individual features still exhibited local fluctuations. To address this, we applied a secondary smoothing process to the extracted feature sequences (*F*_1_ to *F*_6_), again employing the S-G filtering algorithm. This step aims to suppress inter-cycle short-term noise, thereby highlighting the long-term evolutionary trends of the physical characteristics during aging. This provides a clearer and more regular input sequence for the subsequent GRU model. The evolutionary trajectories of the features *F*_1_ - *F*_6_ after S-G smoothing are presented in Fig 4.

To investigate the evolution patterns of extracted health features throughout the battery lifecycle, we visualized the six-dimensional feature vectors for all valid cycles (as shown in Fig 4). Analysis reveals a distinct phase transition in their evolution patterns during the battery lifecycle, occurring around the 32nd cycle.

Initial Stage (Cycles 1–32): All features exhibit highly nonlinear dynamics with significant volatility. Specifically, voltage-coordinate features (*F*_1_*, F*_3_*, F*_5_) exhibit rapid unidirectional changes, while the DTV amplitude-based features (*F*_2_*, F*_4_*, F*_6_) display complex non-monotonic fluctuations characterized by a “first-decrease-then-increase” pattern.

Mid-to-late stage (after 32 cycles): Upon entering this phase, the evolutionary behavior of all features transitions to a more pronounced, gradual, and stable decline pattern. For instance, some voltage features shift to an upward trend, while amplitude features enter a long-term downward trajectory.

This phased evolutionary characteristic observed in the data aligns closely with the initial “break-in” effect or electrochemical “adaptation period” commonly observed in LIBs. From an electrochemical perspective, this phase is primarily driven by the rapid growth and restructuring of the solid electrolyte interphase (SEI) film on the anode surface, accompanied by irreversible consumption of active lithium and dramatic changes in internal resistance [36,37]. This aligns with the nonlinear, abrupt changes in characteristic parameters observed in Fig 4.

The distinctiveness of this initial phase raises a critical question: the impact of atypical data from this stage on long-term stable degradation prediction models remains unclear. Subsequent sections will therefore systematically evaluate the influence of early atypical data on long-term model prediction performance through experiments and validate corresponding data processing strategies.

## 2 PSO-GRU-based SOH estimation method

### 2.1 Gated recurrent unit

**Fig 5:**
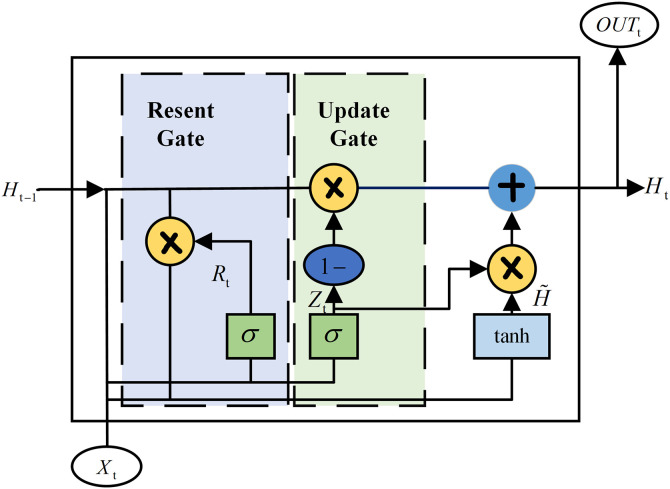
Structure of the GRU.

GRU is a significant variant of Recurrent Neural Networks (RNNs), designed to mitigate the gradient vanishing and exploding issues commonly encountered in traditional RNNs when processing long sequence data [38]. Compared to Long Short-Term Memory (LSTM) networks, GRUs maintain comparable performance while reducing model complexity and computational overhead through a simplified gating mechanism. Its core concept involves introducing two gating vectors—the Reset Gate and the Update Gate—to adaptively control the retention and flow of historical information. Its structural diagram is shown in Fig 5.

The specific computation process of the GRU unit at each time step t is as follows. Its inputs are the current time step’s feature vector *X*_*t*_ and the previous time step’s hidden state $H_{t-\text{1}}$.

#### 1. Reset gate (*R*_*t*_).

The Reset Gate *R*_*t*_ determines how much information from the previous hidden state $H_{t-\text{1}}$ should be “ignored” or “reset.” This enables the model to discard historical information no longer needed for future predictions. Its calculation formula is given by Eq (7):


$$R_{t}=\sigma (X_{t}W_{xr}+H_{t-1}W_{hr}+b_{r})$$ (7)

Where $R_{t}\;$ is the reset gate activation vector at time step *t*, with elements ranging within the interval (0, 1); *X*_*t*_ denotes the input feature vector at time step *t* (in this study, the dimension is 6, corresponding to the six extracted health features);$\;H_{t-\text{1}}$ represents the hidden state vector from the previous time step *t* – 1, encapsulating a summary of historical information; *W*_*xr*_ and *W*_*hr*_ are the weight matrices associated with the input *X*_*t*_ and the previous hidden state $H_{t-\text{1}}$, respectively; *b*_*r*_ is the bias vector for the reset gate; and σ denotes the Sigmoid activation function, which maps any real-valued input to the (0, 1) interval to serve as a gating signal controlling the information flow.

#### 2. Update gate (*Z*_*t*_).

The Update Gate controls the ratio between the inflow of new information and the retention of old information, which is crucial for the long-term memory capability of the GRU. Its calculation is given by Eq (8):


$$Z_{t}=\sigma (X_{t}W_{xz}+H_{t-1}W_{hz}+b_{z})$$ (8)

Where *W*_*xz*_, *W*_*hz*_, and *b*_*z*_ represent the weight matrices and bias vector corresponding to the update gate, respectively.

#### 3. Candidate hidden state (${\tilde{H}}_{t}$).

Based on the filtering results from the reset gate, the model integrates the current input information to compute a temporary candidate hidden state, as defined in Eq (9):


$${\tilde{H}}_{\text{t}}=tanh(X_{t}W_{\text{xh}}+(R_{t}⊙H_{t-1})W_{hh}+b_{h})$$ (9)

where $\;{\tilde{H}}_{t}$ represents the candidate hidden state vector at time step t; *W*_*xh*_, *W*_*hh*_ and *b*_*h*_ denote the weight matrices and bias vector used to compute the candidate hidden state, respectively; ⨀ indicates the Hadamard product (element-wise product); and *tanh* refers to the hyperbolic tangent activation function, which maps input values to the interval (−1, 1).

#### 4. Final hidden state (*H*_*t*_).

Finally, the model linearly combines the previous hidden state $H_{t-\text{1}}$ and the current candidate state ${\tilde{H}}_{t}$ using the update gate *Z*_*t*_ to generate the final output for the current time step. The calculation is expressed in Eq (10):


$$H_{t}=(1-Z_{t})⊙H_{t-1}+Z_{t}⊙{\tilde{H}}_{\text{t}}$$ 10)

This update mechanism balances the trade-off between “forgetting” old information and “remembering” new information via *Z*_*t*_, thereby effectively capturing long-term dependencies in sequence data.

**Fig 6:**
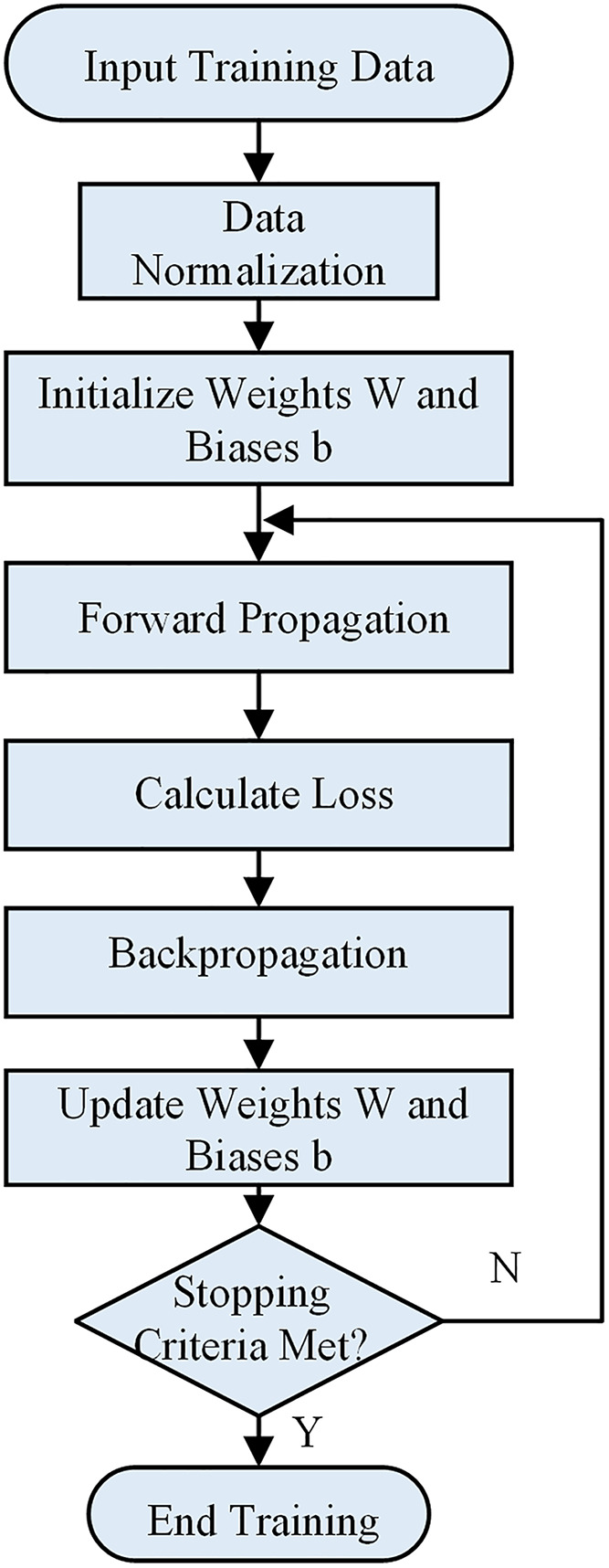
Training process of the baseline GRU model.

The training process of the GRU model is illustrated in Fig 6.

The PSO-GRU model constructed in this paper employs a sequence-to-value regression architecture for temporal SOH prediction. Specifically, at the *t*-th cycle, the model takes the six-dimensional health feature vector [*F*_1_*, F*_2_*, F*_3,_ *F*_4_*, F*_5_*, F*_6_] extracted from that cycle as input for the current time step and outputs an estimated SOH value for that cycle. The recurrent nature of the GRU layer enables it to automatically learn and utilize historical information from the first to the (*t* – 1) th cycle (stored in the hidden state) to assist predictions at the current time step, eliminating the need for manually defining fixed-length time windows. This architecture dynamically captures the degradation trajectory throughout the battery’s entire lifecycle. During model training, we employ the Adam optimization algorithm to update network weights, with its initial learning rate determined through global optimization using the particle swarm optimization algorithm introduced in subsequent sections.

### 2.2 Hyperparameter optimization based on particle swarm optimization

The predictive performance of GRU models is highly dependent on hyperparameter settings. Traditional manual trial-and-error methods struggle to traverse non-convex parameter spaces to find global optima and are inefficient. Therefore, this paper introduces the PSO algorithm to achieve adaptive optimization of key GRU hyperparameters. PSO is an evolutionary computation technique based on swarm intelligence. Through iterative particle search in the solution space, it utilizes individual historical best (*P*_*best*_) and global best (*G*_*best*_) values to guide swarm convergence. Characterized by rapid convergence and strong global search capabilities, PSO is well-suited for solving complex combinatorial optimization problems like neural network hyperparameter tuning.

The hyperparameter optimization strategy within the PSO-GRU framework is designed as follows:

#### 1. Optimization variables and parameter space.

This study jointly optimizes the two hyperparameters most influential to model performance: the number of GRU hidden layer neurons (*N*_*hidden*_) and the initial learning rate (*η*).

*N*_*hidden*_: This parameter determines the model’s fitting capability. Considering that the DTV health feature vectors used in this study underwent physical mechanism screening, resulting in low dimensionality (6 dimensions) and high information density, coupled with the limited sample size of battery aging experimental data, excessive model complexity is highly prone to overfitting. Therefore, a regularization approach is introduced by restricting the search space, setting the search interval for *N*_*hidden*_ to the streamlined range [1,10]. Experiments demonstrate that this range adequately captures the temporal dependencies of the sequence while ensuring the model’s generalization capability on the test set.
*η*: This parameter controls the step size and convergence speed of gradient descent. Its search range is set to [0.001,0.05] to balance training efficiency and avoidance of local optima.

#### 2. Fitness function.

To establish an evaluation criterion for optimization, the fitness function is defined as the root mean square error (RMSE) of the model on the training set. The PSO algorithm aims to find a parameter combination (*N*_*hidden*_*, η*) that minimizes RMSE, thereby ensuring optimal fitting of the model to the aging trajectory.

#### 3. Optimization process.

To mathematically formalize the optimization process, the PSO algorithm initializes a swarm of random particles, where each particle represents a potential hyperparameter combination. In a *D*-dimensional search space (here, *D* = 2), the position of the i-th particle is defined as a vector *X*_*i*_ = [*x*_*i,*1_, *x*_*i,*2_], which specifically corresponds to the hyperparameters being optimized in this study: *X*_*i*_ = [*N*_*hidden*_, *η*].

During the iterative process, the velocity *V*_*i*_ = [*v*_*i,*1_, *v*_*i,*2_] and position *X*_*i*_ of each particle are updated according to the following mathematical expressions:


$$\overset{d}{\underset{i}{v}}(t+1)=w\cdot \overset{d}{\underset{i}{v}}\left(t\right)+c_{\text{1}}r_{\text{1}}\left(Pbes\overset{d}{\underset{i}{t}}-\overset{d}{\underset{i}{x}}\left(t\right)\right)+c_{\text{2}}r_{\text{2}}\left(Gbest^{d}-\overset{d}{\underset{i}{x}}\left(t\right)\right)$$ (11)

$$\overset{d}{\underset{i}{x}}(t+1)=\overset{d}{\underset{i}{x}}\left(t\right)+\overset{d}{\underset{i}{v}}(t+1)$$ (12)

where *t* represents the current iteration step. The terms *w*, *c*_1_, and *c*_2_ are the intrinsic hyperparameters of the PSO model: *w* is the inertia weight controlling the global and local search capabilities; *c*_1_ and *c*_2_ are the cognitive and social learning factors, respectively; *r*_1_ and *r*_2_ are random numbers uniformly distributed in [0,1]. $P\overset{d}{\underset{i}{best}}$ is the historical best position of the *i*-th particle, and $G{best}^{d}$ is the global best position found by the entire swarm.

As previously defined, the fitness function $F(X_{i})$ evaluates the quality of the parameters by calculating the Root Mean Square Error (RMSE) on the training set:


$$F\left(X_{i}\right)=\sqrt{\frac{\text{1}}{N_{train}}\sum_{k=1}^{N_{train}}{\left({\hat{y}}_{k}\left(X_{i}\right)-y_{k}\right)}^{\text{2}}}$$ (13)

where *N*_*train*_ is the number of samples in the training set, *y*_*k*_ is the true SOH value, and ${\hat{y}}_{k}(X_{i})$ specifically denotes the SOH predicted by the GRU model parameterized by the particle vector *X*_*i*_.

Unlike the general RMSE metric used for final model evaluation (which will be detailed in Eq (15)), Eq (13) functions strictly as the dynamic objective function during the PSO search phase. By continuously updating particle velocities and positions to minimize *F*(*X*_*i*_), the algorithm outputs the global optimum solution (G_*best*_) upon reaching the preset iteration count, which optimally configures the final PSO-GRU SOH estimation model.

### 2.3 PSO-GRU-based SOH estimation framework

**Fig 7:**
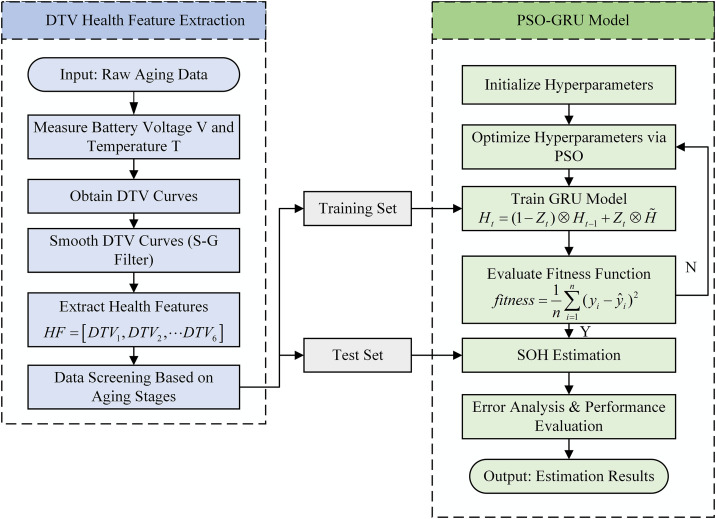
Overall flowchart of the proposed SOH estimation framework.

The PSO-GRU-based SOH estimation workflow, illustrated in Fig 7, primarily comprises the following steps: DTV curve extraction, S-G filter smoothing, feature vector construction, and model prediction.

## 3. Experimental results and analysis

Previous analysis indicates the existence of an atypical early stage in battery lifecycle data, whose impact on long-term prediction models warrants further investigation. To systematically address this question and comprehensively evaluate both the overall performance of the proposed PSO-GRU model and the effectiveness of the data filtering strategy, this chapter designs and implements a two-stage progressive comparative experiment.

### 3.1 Experimental design

To evaluate the performance of the PSO-GRU model and the effectiveness of the data filtering strategy, this chapter designed a phased, progressive comparative experiment. All experiments were conducted on the NASA B0005 dataset. To simulate predictions of future states, the dataset was strictly divided into a training set comprising the first 70% of data in chronological order and an independent test set comprising the remaining 30%. To quantitatively evaluate and compare the predictive performance of each model, this paper employs mean absolute error (MAE), RMSE, and Coefficient of Determination (R-Squared, R²) as evaluation metrics. Their calculation formulas are shown in Equations (14) to (16).


$$\text{MAE}=\frac{\text{1}}{N}\sum_{i=1}^{N}\left|y_{i}-{\hat{y}}_{i}\right|$$ (14)

$$\text{RMSE}=\sqrt{\frac{\text{1}}{N}\sum_{i=1}^{N}(y_{i}-\hat{y_{i}}{)}^{\text{2}}}$$ (15)

$${\text{R}}^{\text{2}}=1-\frac{\sum (y_{i}-{\hat{y}}_{i}{)}^{\text{2}}}{\sum (y_{i}-\overset{―}{y}{)}^{\text{2}}}$$ 16)

Here, *y*_*i*_ denotes the true value, ${\hat{y}}_{i}$ represents the estimated output value, and *N* indicates the number of capacity tests. MAE and RMSE intuitively reflect the absolute magnitude of prediction errors, with smaller values indicating higher model accuracy. R*²* measures the model’s ability to explain data variability, where values closer to 1 signify superior model fit.

**Phase I**: Benchmark model performance evaluation. This phase aims to determine the optimal baseline model architecture. Performance comparisons are conducted between the proposed PSO-GRU model and standard GRU and LSTM models (used as benchmarks) on the standard full-cycle training data (i.e., data encompassing all early cycles).
**Phase II**: validation of optimization strategy effectiveness. After identifying the optimal model (PSO-GRU) in Phase I, this phase verifies the effectiveness of the “early data exclusion” strategy proposed in Chapter 2. We compare the performance of the optimal model under two training strategies:
  Baseline Strategy: Training using full-cycle data (results identical to Phase I).
  Optimized Strategy: Leveraging the distinct characteristics of the early battery aging stage observed in Chapter 2, this strategy evaluates the impact of excluding atypical data from this phase on the model’s long-term prediction performance. Specifically, we removed the first 32 cycles of data from the training set, enabling the model to focus on learning the more universal or regular patterns of stable degradation observed in the mid-to-late stages of battery life.

### 3.2 Benchmark model performance comparison

The first phase of the experiment aimed to select the optimal foundational architecture from three candidate models (GRU, LSTM, PSO-GRU). To this end, all models were trained on the full-cycle dataset (i.e., including early atypical data) under their respective optimal hyperparameter configurations and evaluated on an independent test set. The quantitative evaluation results for each model are shown in Table 1.

**Table 1:** Performance evaluation of different models trained on full-cycle data.

Model	R^2^	MAE (%)	RMSE (%)
GRU	0.6703	4.1359	4.3870
LSTM	0.6394	4.1575	4.4291
PSO-GRU	0.8886	1.4544	1.8587

The quantitative results in Table 1 demonstrate that under identical baseline training conditions, the PSO-GRU model constructed in this paper significantly outperforms standard GRU and LSTM models across all evaluation metrics.

In terms of prediction accuracy, the PSO-GRU model achieves a MAE of 1.45% and a Root RMSE of 1.86%. Compared to the next-best standard GRU model (MAE = 4.14%, RMSE = 4.39%), the PSO-GRU model achieved approximately 64.8% reduction in MAE and 57.6% reduction in RMSE. These results demonstrate that particle swarm optimization of hyperparameters significantly enhances model prediction accuracy.

In terms of trend-fitting capability, the PSO-GRU model also demonstrated a significant advantage. Its coefficient of determination R² reached 0.88855, substantially higher than the 0.67028 achieved by the GRU model and the 0.63943 achieved by the LSTM model. This indicates that the PSO-GRU model more effectively explains the variance in SOH data within the test set, meaning its predicted curve aligns more closely with the overall dynamic trends of the actual data.

**Fig 8:**
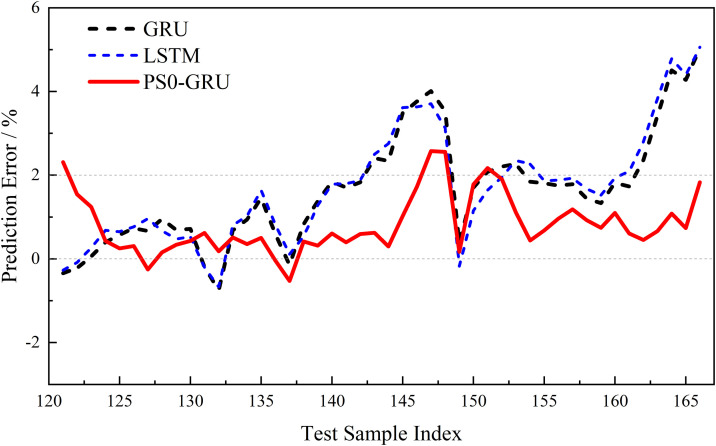
Comparison of prediction errors of different models on the test set.

Fig 8 illustrates the dynamic evolution of prediction errors across different models, further validating the superiority of the PSO-GRU model.

Fig 8 provides further intuitive confirmation of the PSO-GRU model’s superiority from the perspective of dynamic prediction error variation. The figure shows that the error trajectory of the proposed PSO-GRU model (red solid line) exhibits optimal stability throughout the entire test interval. Compared to the benchmark models, which exhibits errors approaching 5% during the later stages of aging, the PSO-GRU model’s prediction error consistently fluctuates around the zero axis. Referencing the 2% tolerance line in the figure, it is evident that the vast majority of the model’s prediction errors are confined within this 2% tolerance range, with only a few isolated data points showing minor overshoots. This demonstrates the model’s excellent stability and robustness.

In stark contrast, the standard GRU (gray dashed line) and LSTM (green dashed line) models exhibit not only larger overall fluctuations in their error curves but also a pronounced divergence trend toward the end of battery life (after sample 155), with maximum prediction errors approaching 5%. This phenomenon indicates that the baseline models suffer from deteriorating prediction performance and insufficient stability when handling the complex nonlinear dynamics during the final stages of battery aging.

In summary, the visualized error analysis results align closely with the quantitative metrics in Table 1 collectively demonstrating that the PSO-GRU model outperforms both standard GRU and LSTM models in both prediction accuracy and robustness. Consequently, PSO-GRU is identified as the optimal baseline model and will be employed for the subsequent phase of optimization strategy validation.

### 3.3 Analysis of optimization strategy effectiveness

After confirming PSO-GRU as the optimal base model, the experiment proceeded to the second phase to validate the effectiveness of the “early data exclusion” strategy. Table 2 compares the performance of the PSO-GRU model under the baseline strategy (using full-cycle data) and the optimization strategy (excluding early data).

**Table 2:** Impact of training data strategy on PSO-GRU model performance.

Model	R^2^	MAE (%)	RMSE (%)
Benchmark Strategy	0.8886	1.4544	1.8587
Optimization Strategy	0.9165	0.7462	0.9660

The quantitative results in Table 2 clearly demonstrate that the data filtering strategy significantly enhances model performance. Compared to the baseline strategy, the optimized strategy reduces the model’s MAE from 1.45% to 0.75%, representing a 48.7% decrease; the Root RMSE decreases from 1.86% to 0.97%, achieving a 48.1% reduction. Simultaneously, the coefficient of determination R² improved from 0.8886 to 0.9165.

**Fig 9:**
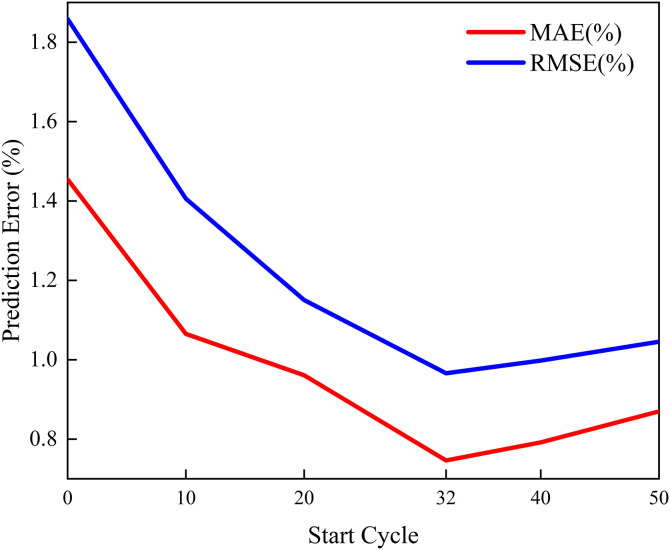
Analysis of the Impact of Initial Loop Points on Model Prediction Errors (MAE and RMSE).

To further quantitatively validate the rationality of setting the cutoff point at 32 iterations, this section conducts a sensitivity analysis experiment on the starting iteration point. While keeping the test set unchanged, we set the starting point of the training set to 0, 10, 20, 32, 40, and 50 respectively, and recorded the changes in MAE and RMSE of the model on the test set. The results are shown in Fig 9.

As shown in Fig 9, both the model’s prediction errors (MAE and RMSE) exhibit a significant downward trend as the starting point of the cycle is shifted backward (from 0 to 32). This confirms that including early non-stationary “formation period” data does indeed introduce noise, interfering with the model’s learning of the long-term decline trend. When the starting point is set to 32, both metrics reach their lowest values (MAE = 0.75%, RMSE = 0.97%). However, it is noteworthy that when the cutoff point was further increased to 40 and 50, the errors did not continue to decrease but instead slightly rebounded. This indicates that while noise removal is crucial, excessively reducing the training data volume also weakens the model’s generalization capability. Therefore, selecting 32 cycles as the cutoff threshold achieves the optimal balance between “noise removal” and “preserving effective information.”

**Fig 10:**
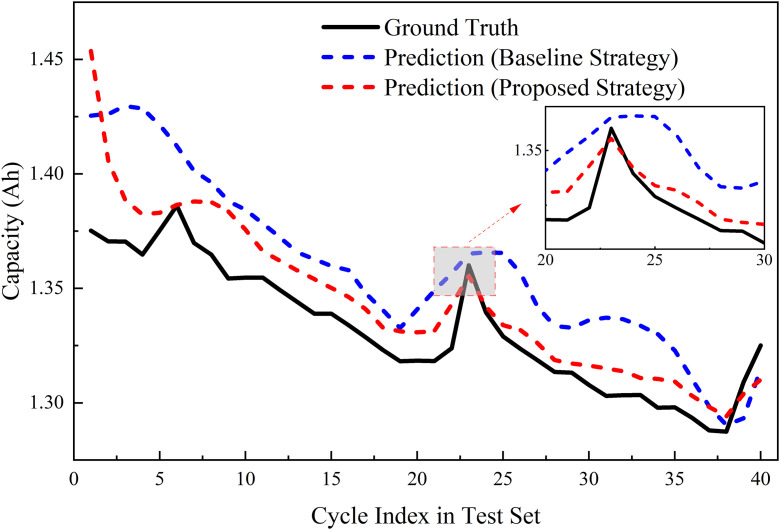
Prediction comparison of PSO-GRU model under two training strategies.

Fig 10 visually illustrates this performance enhancement.

As observed in Fig 10: The model trained with the baseline strategy (blue dashed line) generally reflects the overall degradation trend in its predicted trajectory. However, it exhibits a persistent and significant systematic deviation from the true capacity curve (black solid line). This deviation is particularly pronounced in regions where the battery exhibits complex dynamics such as localized capacity recovery (e.g., near cycle number 23, as detailed in the zoomed-in view). Within this interval, the baseline model fails to capture this non-monotonic change, continuing its previous fading trend and resulting in substantial local prediction errors.

In contrast, the model trained with the optimization strategy (red solid line) demonstrates markedly improved prediction accuracy. Its predicted trajectory closely aligns with actual values across the entire test set. Notably, as shown in the zoomed-in subplot, this model successfully reproduces the phenomenon of local capacity recovery within the overall decline trend, indicating its ability to learn and generalize the battery’s inherent complex nonlinear degradation behavior.

In summary, both quantitative evaluation results and visual analysis provide robust support for the data filtering strategy proposed in this paper: selectively removing early-stage atypical data from the training set guides the model to focus on learning the more representative aging patterns observed in the mid-to-late stages of battery life. This approach not only enhances the model’s accuracy in predicting overall degradation trends but also strengthens its ability to capture critical nonlinear dynamics such as capacity recovery, ultimately achieving a substantial improvement in predictive performance.

### 3.4 Validation of generalization on other battery datasets

To further demonstrate the robustness, generalization capability, and novelty of the DTV feature extraction and PSO-GRU model proposed in this paper, and to avoid the model overfitting a single dataset (B0005), this section introduces another independent dataset—the NASA B0007 battery dataset—for cross-validation. The B0007 battery underwent aging tests at the same room temperature (24°C) as the B0005 battery but exhibited a slightly different capacity decay trajectory, providing a rigorous test of the model’s adaptability.

In this validation experiment, we applied the exact same DTV feature extraction process and PSO-GRU hyperparameter optimization framework to the B0007 dataset as described earlier. As before, the first 70% of the lifespan data in chronological order was used for model training, while the remaining 30% served as a completely independent test set.

The experimental results demonstrate that our model maintains extremely high prediction accuracy on the new dataset. Specifically, the model achieved an MAE of 0.9869% and an RMSE of 1.4024% on the B0007 dataset.

**Fig 11:**
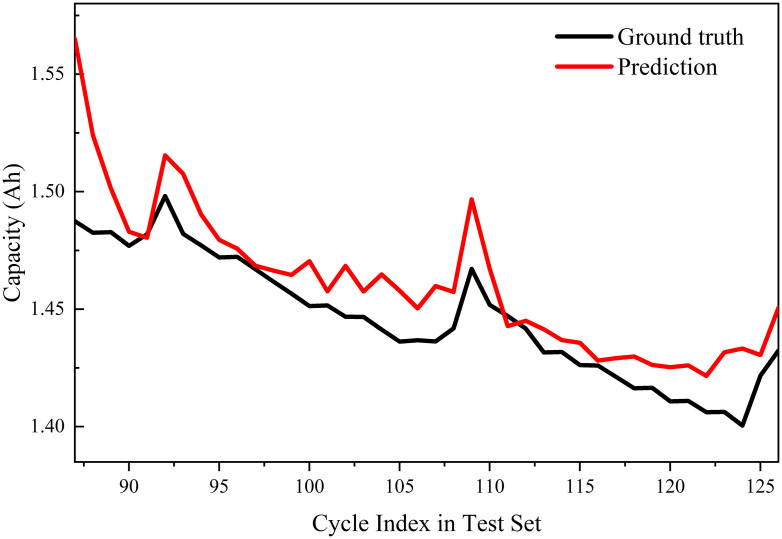
SOH estimation results of the proposed PSO-GRU model on the NASA B0007 dataset.

The SOH prediction trajectory for the B0007 dataset is shown in Fig 11. As can be clearly seen from the figure, the prediction curve generated by the PSO-GRU model closely tracks the actual capacity degradation trajectory. These cross-dataset validation results strongly demonstrate that the method proposed in this paper is not limited to a specific individual battery but possesses excellent cross-dataset generalization capabilities, further highlighting the novelty and practical value of this framework in real-world battery health management.

### 3.5 Discussion: practical significance of data-driven strategy and model generalization capability

The aforementioned experimental results collectively reveal the comprehensive advantages of the proposed framework and confirm the importance of data filtering strategies in SOH estimation. The underlying mechanisms and value analysis are as follows.

First, from a model architecture perspective, the superiority of PSO-GRU stems from its effective hyperparameter optimization capability. The performance of standard GRU and LSTM models heavily relies on manually set parameters. In contrast, our experiments demonstrate that global search via the particle swarm optimization algorithm can identify a significantly superior hyperparameter combination for the model, particularly in capturing data dynamic trends (*R*²) and suppressing late-stage error divergence.

Second, from a data strategy perspective, the core finding—that excluding early data significantly enhances estimation performance—holds profound scientific implications and engineering practical value.

From an electrochemical perspective, the early cycling phase of a battery represents a non-stationary “adaptation period” or “formation phase.” During this stage, the internal electrochemical environment undergoes rapid changes, with the accelerated growth of the SEI film causing a swift and nonlinear capacity decline. This initial degradation pattern can vary considerably across different batches or even individual batteries, lacking representative characteristics. Incorporating these data into training risks model overfitting to transient, unstable decay patterns, compromising its predictive capability for the subsequent, longer-lasting stable decay phase and ultimately degrading generalization performance. Thus, strategically excluding this phase’s data fundamentally prevents the model from being misled by “out-of-distribution” data from the initial stage.

At the engineering practice level, this strategy aligns closely with the core application scenarios of BMS. The primary mission of BMS is to provide precise early warnings for mid-to-late-stage battery health, with its core tasks being accurate remaining useful life (RUL) forecasting and preventing safety incidents. During the initial purchase and usage phase, when battery capacity remains close to nominal values, subtle capacity fluctuations are typically not a primary concern for users nor the core basis for BMS lifespan warnings. Consequently, a prediction model focused on the stable degradation phase in the mid-to-late stages holds greater practical application value. By concentrating on this critical phase, the approach outlined in this paper provides more reliable decision support for BMS, which is crucial for enhancing system safety and reliability.

## 4. Conclusion

This paper addresses the lack of physical interpretability and weak generalization capabilities in purely data-driven methods for estimating SOH of LIBs. It proposes and validates a comprehensive modeling framework that integrates physical features, intelligent optimization algorithms, and innovative data strategies. First, the DTV method is employed to extract health features with explicit physical significance. The PSO-GRU model based on these features demonstrates significantly superior prediction accuracy and stability compared to standard GRU and LSTM models.

The primary contribution lies in proposing and validating an optimized training strategy that “excludes early non-typical data.” Experimental results demonstrate that this strategy enables a qualitative leap in the PSO-GRU model’s predictive performance, reducing both MAE and RMSE by nearly 50%. This finding reveals the critical importance of strategically filtering training data when handling industrial time-series data with an initial adaptation phase for constructing high-performance predictive models.

Furthermore, the “gray-box” framework constructed herein provides an effective approach to addressing the interpretability issue of data-driven models. Rather than completely abandoning neural networks, it transforms the GRU’s learning task from “blindly searching for patterns in raw data” to “fitting the evolutionary trends of known physical features” by incorporating DTV features derived from physical mechanisms as model inputs. Unlike traditional data-driven models that utilizing raw voltage and temperature sequences as independent inputs—thereby overlooking their intrinsic thermo-electric coupling—DTV analysis mathematically forces a correlation between thermal response and voltage changes. This effectively characterizes the entropy-heat generation rate associated with electric potential. As demonstrated by the feature extraction results, DTV peaks align more distinctly with phase transition points than raw temperature curves, delivering higher signal-to-noise ratios while filtering out non-aging thermal fluctuations caused by environmental conditions. This physics-information fusion paradigm significantly enhances model decision transparency and reliability while maintaining high accuracy.

Although the framework proposed in this paper demonstrates excellent predictive performance on both B0005 and B0007 batteries, it still has certain limitations. First, although cross-battery validation was conducted, the experimental data are primarily limited to the same chemical system (e.g., lithium cobalt oxide) and laboratory-controlled, constant-temperature conditions. Battery aging pathways may differ across different chemical systems (e.g., NCM, LFP) or under complex dynamic conditions (e.g., real-world driving conditions for electric vehicles). Therefore, the method’s generalization capability on broader cross-system datasets and the universal threshold setting for the “early data removal” strategy require further cross-validation. Secondly, the effective extraction of DTV features relies on high-quality surface temperature measurements. In practical applications, significant sensor noise or substantial thermal hysteresis may compromise feature fidelity and model accuracy. Future work will focus on extending this framework to more battery samples and exploring adaptive data truncation algorithms to enhance the method’s robustness and engineering utility.

In summary, this paper not only presents a high-performance SOH prediction model but also offers novel insights and methodologies for developing reliable, application-oriented predictive models for battery management systems and other fields.
